# Spatiotemporal Transcriptome Analysis Provides Insights into Bicolor Tepal Development in *Lilium* “Tiny Padhye”

**DOI:** 10.3389/fpls.2017.00398

**Published:** 2017-03-24

**Authors:** Leifeng Xu, Panpan Yang, Yayan Feng, Hua Xu, Yuwei Cao, Yuchao Tang, Suxia Yuan, Xinyan Liu, Jun Ming

**Affiliations:** ^1^Key Laboratory of Biology and Genetic Improvement of Horticultural Crops, Ministry of Agriculture, Institute of Vegetables and Flowers, Chinese Academy of Agricultural SciencesBeijing, China; ^2^Department of Ornamental Plants, College of Landscape Architecture, Nanjing Forestry UniversityNanjing, China

**Keywords:** *Lilium* spp., transcriptome, bicolor tepals, anthocyanin biosynthesis, chlorophyll metabolism, transcriptional network

## Abstract

The bicolor Asiatic hybrid lily cultivar “Tiny Padhye” is an attractive variety because of its unique color pattern. During its bicolor tepal development, the upper tepals undergo a rapid color change from green to white, while the tepal bases change from green to purple. However, the molecular mechanisms underlying these changes remain largely uncharacterized. To systematically investigate the dynamics of the lily bicolor tepal transcriptome during development, we generated 15 RNA-seq libraries from the upper tepals (S2-U) and basal tepals (S1-D, S2-D, S3-D, and S4-D) of *Lilium* “Tiny Padhye.” Utilizing the Illumina platform, a total of 295,787 unigenes were obtained from 713.12 million high-quality paired-end reads. A total of 16,182 unigenes were identified as differentially expressed genes during tepal development. Using Kyoto Encyclopedia of Genes and Genomes pathway analysis, candidate genes involved in the anthocyanin biosynthetic pathway (61 unigenes), and chlorophyll metabolic pathway (106 unigenes) were identified. Further analyses showed that most anthocyanin biosynthesis genes were transcribed coordinately in the tepal bases, but not in the upper tepals, suggesting that the bicolor trait of “Tiny Padhye” tepals is caused by the transcriptional regulation of anthocyanin biosynthetic genes. Meanwhile, the high expression level of chlorophyll degradation genes and low expression level of chlorophyll biosynthetic genes resulted in the absence of chlorophylls from “Tiny Padhye” tepals after flowering. Transcription factors putatively involved in the anthocyanin biosynthetic pathway and chlorophyll metabolism in lilies were identified using a weighted gene co-expression network analysis and their possible roles in lily bicolor tepal development were discussed. In conclusion, these extensive transcriptome data provide a platform for elucidating the molecular mechanisms of bicolor tepals in lilies and provide a basis for similar research in other closely related species.

## Introduction

Lily (*Lilium* spp.) is one of the most important ornamental plants because of their large flowers with unique and diverse colors. Hybrid lily cultivars are grouped according to their genetic phylogeny (Shahin et al., 2014). Asiatic hybrids, one of the major groups of hybrids, are derived from interspecific crosses among species in the section Sinomartagon that are mainly distributed in China. A typical ornamental feature of Asiatic hybrid lilies is the variety of flower colors, including yellows, oranges, pinks, reds, and whites. In addition to the various colors, Asiatic hybrid lilies also exhibit variations in pigmentation patterns, including spots and bicolors.

Bicolor flowers have fascinating color patterns. Some of the molecular mechanisms leading to the development of bicolor petals have been identified. Post-transcriptional gene silencing (PTGS) of *chalcone synthase* (*CHS*) genes in non-pigmented areas produces the white areas of bicolor flower petals in several horticultural crops, such as petunia (*Petunia hybrida*) (Koseki et al., 2005; Saito et al., 2006; Morita et al., 2012), camellia (*Camellia japonica*) (Tateishi et al., 2010), and dahlia (*Dahlia variabilis*) (Ohno et al., 2011). Two types of bicolor flowers are found in Asiatic hybrid lilies; in one type, anthocyanins accumulate in the upper tepals (e.g., “Lollipop”) while in the other type, anthocyanin pigments accumulate in the tepal bases (e.g., “Tiny Padhye”). Recently, Suzuki et al. (2016) showed that the first type of bicolor tepals (“Lollipop”) resuled from the transcriptional regulation of anthocyanin biosynthetic genes. However, whether the same molecular mechanisms underlie the second type of bicolor tepals remains unclear.

Anthocyanins, a class of flavonoid compounds, are responsible for the pink, red, blue, and purple colors in diverse plant tissues (flowers, fruits, leaves, etc.) (Davies et al., 2012; Zhao and Tao, 2015). The anthocyanin biosynthetic pathway is one of the best-known specialized metabolic pathways and most of the anthocyanin biosynthetic genes have been characterized in different plant species (Winkel-Shirley, 2001; Grotewold, 2006). There have been several reports on the molecular mechanisms of the anthocyanin biosynthetic pathway in lilies. Three *CHS* genes (*LhCHSA, LhCHSB*, and *LhCHSC*), *LhDFR, LhPAL, LhF3H, LhF3*′*H, LhANS*, and two R2R3-MYB transcription factors (TFs; *LhMYB6* and *LhMYB12*) were isolated from the tepals of Asiatic lily “Montreux” (Nakatsuka et al., 2003; Yamagishi et al., 2010; Lai et al., 2012). *LhMYB12-Lat* and *LrMYB15* were shown to determine the unique anthocyanin color patterns of the Tango Series cultivars of Asiatic hybrid lilies (Yamagishi et al., 2014) and *Lilium regale* (Yamagishi, 2016), respectively. However, there is still limited information on the overall molecular mechanisms underlying tepal pigmentation in lilies.

The petals of some flowering plants contain chlorophyll (Chl) in the early developmental stages. As the petals mature, the Chl content gradually decreases during the late developmental stages (Ohmiya et al., 2014). The Chl metabolic pathway is relatively well-characterized in many plant species. This pathway has three phases: Chl a synthesis, interconversion of Chl a and Chl b (Chl cycle), and Chl degradation (Eckhardt et al., 2004; Hörtensteiner, 2013). The molecular mechanisms of Chl metabolism in leaves and fruit have been largely unraveled (Lim, 2003; Lai et al., 2015; Wen et al., 2015). Ohmiya et al. (2014) showed that low rates of Chl biosynthesis and high rates of Chl degradation led to the absence of Chls in non-green carnation petals. However, the molecular mechanisms regulating Chl metabolism in lily petals are still unknown.

Whole-transcriptome sequencing based on next-generation sequencing has become a powerful tool to identify candidate genes and investigate gene expression patterns of non-model organisms without reference sequences (Mutz et al., 2013). Furthermore, transcriptome data can be used to identify candidate genes relevant to a given pathway or phenotype using a weighted gene co-expression network analysis (WGCNA). WGCNA is a powerful approach for finding clusters (modules) of highly correlated genes with a high topological overlap (Langfelder and Horvath, 2008; Zhao et al., 2015). This strategy has been used to identify regulators and co-expression networks in *Arabidopsis thaliana* (Appel et al., 2014), strawberry (*Fragaria* spp.) (Hollender et al., 2014), apple (*Malus* × *domestica*) (Bai et al., 2015), and sweet orange (*Citrus sinensis* L. Osbeck) (Huang et al., 2016).

In this study, RNA samples from the upper tepals (stage 2; S2) and tepal bases (S1–4) of “Tiny Padhye” bicolor tepals were sequenced using the Illumina sequencing platform. Global gene expression profiles, focusing mainly on genes in the anthocyanin biosynthetic pathway and Chl metabolic pathway, during bicolor tepal development were analyzed using a differential gene expression strategy and quantitative real-time PCR (qRT-PCR) analyses. We aimed to identify structural genes and TFs associated with the anthocyanin biosynthetic pathway and Chl metabolic pathway, and to investigate their spatiotemporal expression patterns during bicolor tepal development to characterize the mechanisms of bicolor (white and purple) tepals development in lilies.

## Materials and methods

### Plant materials

**Figure 1:**
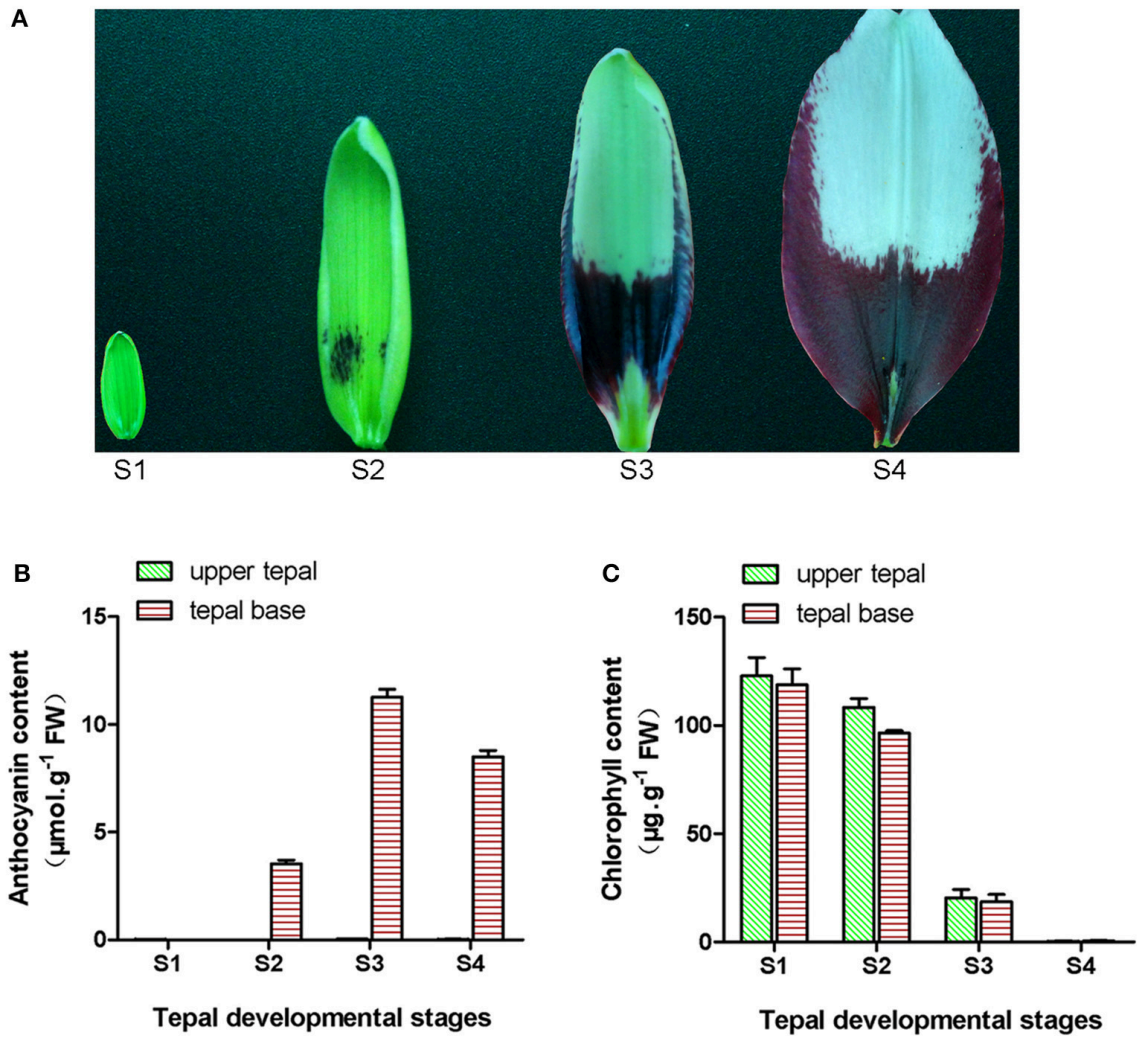
**Phenotype and pigment accumulation in inner tepals of Asiatic “Tiny Padhye” at different developmental stages**. **(A)** Inner tepals of Asiatic “Tiny Padhye” at four tepal developmental stages. **(B)** Total anthocyanin content in lily inner tepals at different developmental stages. **(C)** Total Chl content in lily inner tepals at different developmental stages. Error bars show standard error (SE) of the mean.

The lily Tango Series cultivar “Tiny Padhye” was grown in a greenhouse at the Chinese Academy of Agricultural Sciences (Beijing, China). Flowers sampling was performed at four different developmental stages (Figure 1A): Stage 1 (S1; bud length ~1.5 cm, and no anthocyanins visible on tepals), stage 2 (S2; anthocyanins visible on tepal bases), stage 3 (S3; 1 day before anthesis where the lower half of tepals was fully pigmented), and stage 4 (S4; 0 days post-anthesis). At each stage, samples were taken from the upper inner tepals of 20 flowers and pooled together as one biological sample; three independent biological replicates were collected for each stage. This process was repeated for the inner tepal bases. The collected samples were immediately frozen in liquid nitrogen and then stored at −80°C until use.

### Anthocyanin and chlorophyll assays

Frozen lily tepals were ground into powder in liquid nitrogen. Anthocyanins were extracted with a solvent mixture of trifluoroacetic acid, methanol, methanoic acid, and water (1:70:2:27, v:v:v:v). The extract was then analyzed by high-performance liquid chromatography (HPLC) on a Waters 2695 Alliance HPLC instrument (Waters Corporation, Milford, MA, USA) connected to a Waters 2998 photodiode array detector. The separation was performed on a Waters SunFire C18 column (150 × 4.6 mm I.D., 5 μm) with a C18 guard column at 30°C. Products were detected at 530 nm. Anthocyanins were eluted with a gradient mobile phase consisting of solvent A (HCOOH:H_2_O = 0.1:100, v:v) and solvent B (HCOOH:CH_3_CN = 0.1:100, v:v) at a flow rate of 1.0 ml·min^−1^. The solvent gradient program is shown in Table S1. Six authentic standards [cyanidin (Cy), pelargonidin 3,5-diglucoside (Pg3,5dG), cyanidin 3-O-β-glucoside (Cy3G), cyanidin 3-O-β-rutinoside (Cy3R), and peonidin 3-O-β-glucoside (Pn3G)] were used to identify peaks. Chl content was determined by measuring the absorbance of the supernatant at 663 and 645 nm according to the protocol of Arnon (1949).

### cDNA library construction, sequencing, and transcriptome assembly

Total RNA was extracted from each sample using an RNAprep pure Plant Kit (Tiangen Biotech Co., Ltd., Beijing, China), according to the manufacturer's instructions. Total RNA was extracted from upper tepals at stage 2 (S2-U) and from basal tepals at the four different stages (S1-D, S2-D, S3-D, and S4-D). Three biological replicates were used for each sample. A total of 15 RNA-seq libraries were constructed from these samples using a ScriptSeq mRNA-Seq Library Preparation Kit (Epicentre Biotechnologies, Madison, WI, USA) according to the manufacturer's protocol. The libraries were sequenced to generate 150 paired-end raw reads on an Illumina Hiseq 4000 platform. After clean reads were generated by removing adapters, low-quality reads, and ambiguous reads from the raw data, transcriptome assembly was accomplished using Trinity software as previously described for *de novo* transcriptome assembly without a reference genome (Grabherr et al., 2011).

### Functional annotation and classification

The assembled unigenes were searched against six public databases, including the NCBI Non-Redundant Protein Sequences (NR) database, NCBI Nucleotide Sequences (NT) database, Protein Family (PFAM) database, Swiss-Prot protein database, Gene Ontology (GO) database, Eukaryotic Ortholog Groups (KOG) database, and Kyoto Encyclopedia of Genes and Genomes (KEGG) database.

### Differentially expressed genes analysis

The differentially expressed genes (DEGs) between pairs of samples were identified and filtered with the R package DESeq (Anders and Huber, 2010). The DEGs between two samples were determined based on |log2 (foldchange)| > 1 and padj < 0.05. The heatmap displays of the Trimmed Mean of M-values (TMM) normalized against the Fragment Per Kilobase of transcripts per Million mapped reads (FPKM) were performed created using the R package pheatmap (https://cran.r-project.org/src/contrib/Archive/pheatmap/) (Kolde, 2012).

### qRT-PCR analyses

A total of 10 unigenes related to anthocyanin biosynthesis and nine unigenes related to Chl metabolism were chosen for qRT-PCR analyses. qRT-PCR analyses were performed using SYBR® Premix Ex Taq™ II (Tli RNaseH Plus) (Takara, Dalian, China) and a Bio-Rad iQ5 Gradient RT-PCR system with the following reaction conditions: Denaturation at 95°C for 30 s and 40 cycles of amplification (95°C for 5 s, 60°C for 30 s). The lily *Actin* gene was used as an internal control for normalization. Relative expression levels of target genes were calculated using the 2^−ΔΔCT^ method (Livak and Schmittgen, 2001) against the internal control. The gene-specific primers are shown in Table S2. Experiments were performed with three independent biological replicates and three technical replicates.

### Transcription factor identification

To identify TFs, assembled unigenes were searched against the Plant Transcription Factor Database PlnTFDB (http://plntfdb.bio.uni-potsdam.de/v3.0/downloads.php) using BLASTX with an E-value cut-off of ≤ 10^−5^.

### Gene co-expression network construction and visualization

To investigate the co-expressed gene networks in tepal development with a particular focus on the transcriptional architecture of anthocyanin biosynthesis and Chl metabolism, we constructed gene co-expression networks from the DEGs using the WGCNA package (Langfelder and Horvath, 2008). The networks were visualized using Cytoscape _v.3.0.0 (Hollender et al., 2014).

## Results

### Anthocyanin and chlorophyll levels in upper tepals and tepal bases

During bicolor tepal development in Asiatic “Tiny Padhye,” the upper tepals underwent a rapid color change from green to white, whereas the tepal bases changed from green to purple (Figure 1A). To investigate these physiological changes, the anthocyanin, and Chl contents of upper and lower tepals were assessed at four different stages of tepal development.

Using HPLC, a single anthocyanin (cyanidin 3-O-β-rutinoside) was detected in the pigmented tepal bases of “Tiny Padhye” (Figure S1). No anthocyanins were detected in the upper tepals at any stage of tepal development (Figure 1B). The anthocyanin levels in tepal bases increased dramatically from S1 to S3, and then gradually decreased at S4 (Figure 1B). As shown in Figure 1C, Chls accumulated at the early developmental stages (S1 and S2) in the upper and lower tepal parts before decreasing to extremely low levels at the late stages (S3 and S4).

### Sequencing and sequence assembly

The transcriptomes of the 15 “Tiny Padhye” samples were separately obtained using Illumina technology. Overall, ~772.62 million 150-nt paired-end raw reads were generated (Table 1). All raw reads have been deposited in the NCBI Sequence Reads Archive (SRA) under the accession number SRP093907. After removing low-quality sequences, adapters, and ambiguous reads, we obtained a total of 713.12 million high-quality clean reads (Table 1). These clean reads were assembled into 400,850 transcripts with a mean length of 668 nt and an N50 length of 1,043 nt (Table 1). These transcripts were then assembled into 295,787 unigenes with an average length of 544 nt and an N50 length of 718 nt (Table 1).

**Table 1:** **Summary of transcriptome sequencing data and transcriptome assembly**.

**Sample**	**Raw read**	**Clean read**	**Clean base (G)**	**Error (%)**	**Q20 (%)**	**Q30 (%)**
S1-D-1	44468792	42156576	6.32	0.03	94.08	86.6
S1-D-2	47881358	45402742	6.81	0.03	93.81	86.05
S1-D-3	54186062	51397354	7.71	0.03	93.96	86.24
S2-D-1	44234498	41934920	6.29	0.03	93.81	85.79
S2-D-2	53374880	51428796	7.71	0.02	95.16	88.57
S2-D-3	48243060	45806832	6.87	0.02	95.31	88.82
S2-U-1	64528168	61264224	9.19	0.03	94.6	87.91
S2-U-2	59080054	56621132	8.49	0.03	94.67	88.06
S2-U-3	44247506	42353756	6.35	0.03	94.31	87.33
S3-D-1	47200892	44991602	6.75	0.03	94.59	87.8
S3-D-2	44741180	42851948	6.43	0.03	94.57	87.68
S3-D-3	52893548	50065124	7.51	0.03	94.61	87.82
S4-D-1	52595616	41996740	6.3	0.03	94.98	86.77
S4-D-2	51700800	42999704	6.45	0.03	96.28	89.77
S4-D-3	63243588	51849098	7.78	0.03	96.05	89.26
Total	772620002	713120548				
	**Transcript**	**Unigene**				
Total number	400850	295787				
Total length (nt)	267712500	160816025				
Mean length (nt)	668	544				
Minimum length (nt)	201	201				
Maximum length (nt)	15706	15706				
N50	1043	718				

### Functional annotation and classification

**Figure 2:**
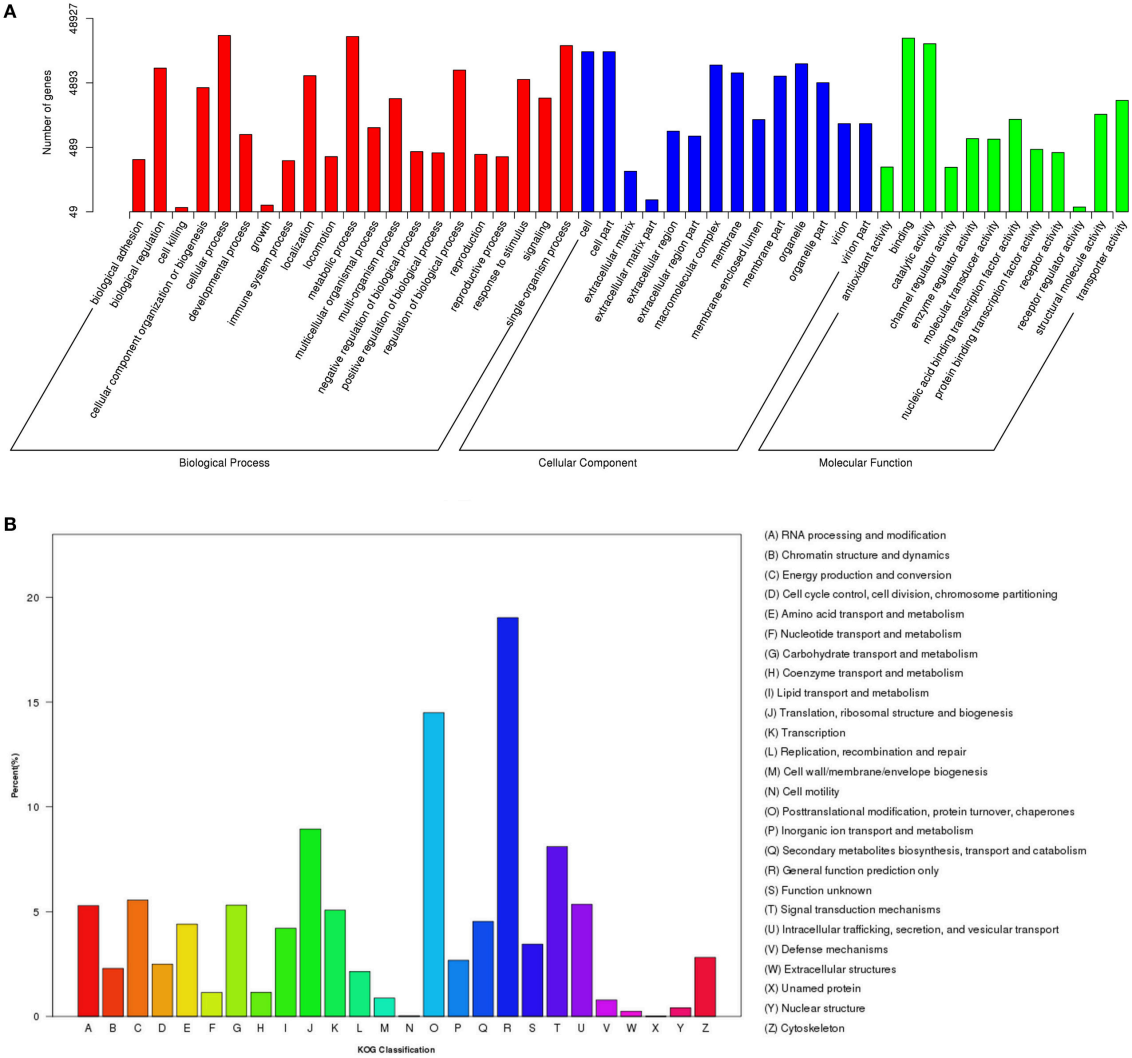
**Functional classification of differentially expressed genes (DEGs) among different samples. (A)** GO functional classification of DEGs. **(B)** KOG functional classification of DEGs.

To annotate the transcriptome with putative functions, the assembled unigenes were searched against four public databases: NR, NT, PFAM, and SWISS-PROT. Among them, 66,531, 25,508, 47,404, and 40,271 unigenes were annotated to the NR database, NT, PFAM, and SWISS-PROT databases, respectively (Table S3). To further illustrate the main biological functions of the transcriptome, GO, KOG, and KEGG pathway analyses were performed. GO analysis provides a description of gene products in terms of their associated Biological Process (BP), Cellular Component (CC), and Molecular Function (MF) (Berardini et al., 2004) (Figure 2A). A total of 48,927 unigenes were categorized into 47 major functional groups. Cellular process (GO:0009987), cell (GO:0005623), and binding (GO:0005488) were the most highly represented GO terms in BP, CC, and MF, respectively (Table S3 and Figure 2A).

In addition to the GO analysis, KOG analysis was performed to further evaluate the function of the assembled unigenes. A total of 17,714 annotated unigenes were grouped into 26 KOG classifications (Table S3 and Figure 2B). The three most abundant groups represented in the transcriptome were group J (translation, ribosomal structure, and biogenesis), group O (post-translational modification, protein turnover, and chaperones) and group R (general function prediction only) (Figure 2B).

In the KEGG pathway analysis, a total of 18,675 unigenes were mapped to 278 KEGG pathways (Table S3). The pathways with the highest unigene representations were ribosome (ko03010, 860 unigenes), followed by carbon metabolism (ko01200, 828 unigenes), and biosynthesis of amino acids (ko01230, 723 unigenes). Notably, 61 unigenes were involved inanthocyanin biosynthesis and 106 unigenes were involved in Chl metabolism (Table 2).

**Table 2:** **Candidate unigenes involved in anthocyanin biosynthesis and chlorophyll metabolism in transcriptome of “Tiny Padhye”**.

**KEGG pathway**	**Gene**	**Protein**	**No. All^a^**	**No. DEGs^b^**
Anthocyanin biosynthesis	*CHS*	Chalcone synthase	41	13
	*CHI*	Chalcone isomerase	1	1
	*F3H*	Flavanone 3-hydroxylase	2	1
	*F3′H*	Flavonoid 3′-hydroxylase	4	1
	*DFR*	Dihydroflavonol 4-reductase	3	1
	*ANS*	Leucoanthocyanidin dioxygenase	3	0
	*UFGT*	Anthocyanidin 3-O-glucosyltransfersae	6	3
	*3RT*	Anthocyanidin-3-glucoside rhamnosyltransferase	1	1
Chlorophyll biosynthesis	*GluRS*	Glutamyl-tRNA synthetase	9	0
	*HEMA*	Glutamyl-tRNA reductase	6	1
	*GSA*	Glutamate-1-semialdehyde 2,1-aminomutase	4	2
	*HEMB*	Porphobilinogen synthase	5	2
	*HEMBS*	Hydroxymethylbilane synthase	2	1
	*HEMD*	Uroporphyrinogen-III synthase	2	1
	*HEME*	Uroporphyrinogen decarboxylase	7	2
	*HEMF*	Coproporphyrinogen III oxidase	6	1
	*HEMG*	Oxygen-dependent protoporphyrinogen oxidase	4	1
	*CHLD*	Magnesium chelatase subunit D	3	1
	*CHlI*	Magnesium chelatase subunit I	4	1
	*CHlH*	Magnesium chelatase subunit H	11	0
	*CHlM*	Magnesium-protoporphyrin O-methyltransferase	3	1
	*CRD*	Magnesium-protoporphyrin IX monomethyl ester (oxidative) cyclase(oxidative) cyclase	6	2
	*POR*	Protochlorophyllide reductase	6	2
	*DVR*	Divinyl chlorophyllide a 8-vinyl-reductase	1	1
	*ChlG*	Chlorophyll synthase	2	0
Chlorophyll cycle	*CAO*	Chlorophyllide a oxygenase	7	0
	*NYC1*	Chlorophyll(ide) b reductase	5	2
	*HCAR*	7-hydroxymethyl chlorophyll a reductase	4	0
Chlorophyll degradation	*CLH*	Chlorophyllase	2	2
	*PPH*	Pheophytinase	3	2
	*PaO*	Pheophorbide a oxygenase	2	1
	*RCCR*	Red chlorophyll catabolite reductase	1	1
	*SGR*	STAY-GREEN	1	1

a *Total number of unigenes analyzed*.

b *Number of differentially expressed genes between samples*.

### Identification of differentially expressed genes

**Figure 3:**
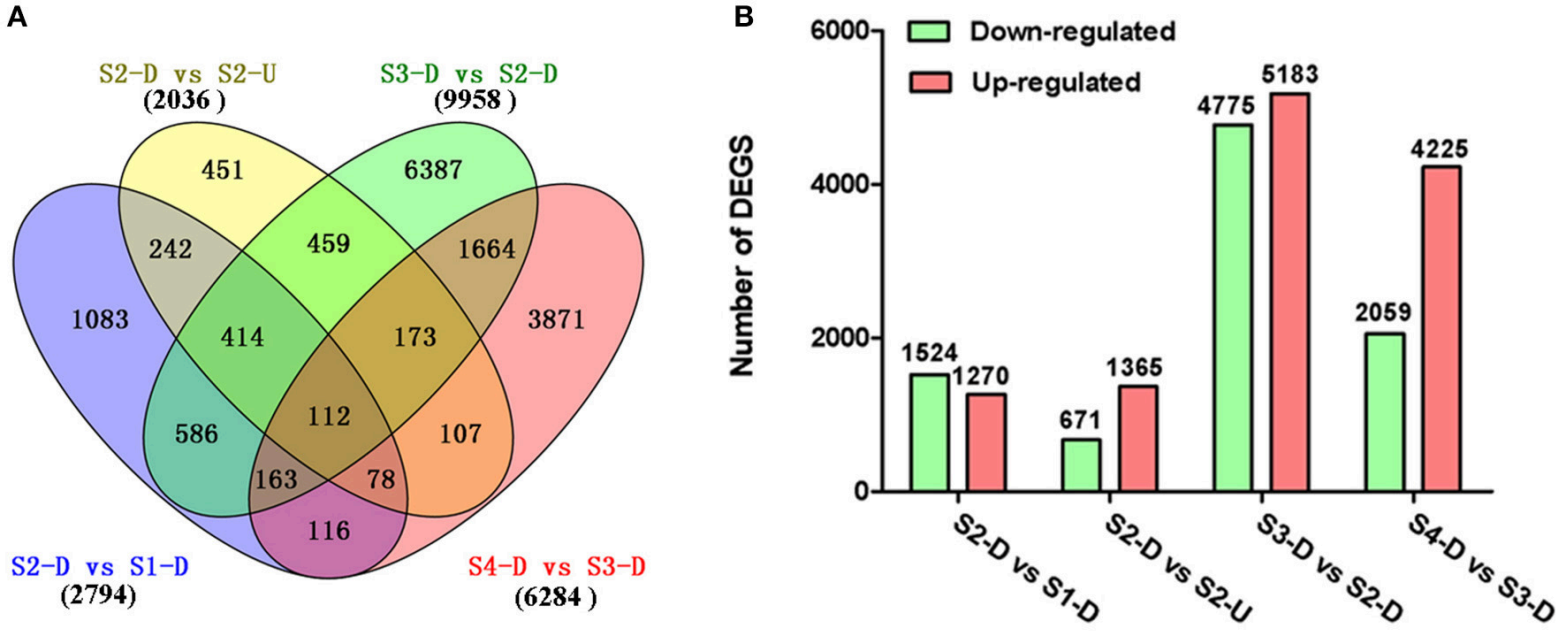
**Distribution of differentially expressed genes (DEGs) in different pair-wise comparisons**. **(A)** Venn diagram illustrating the number of DEGs revealed by paired comparisons. **(B)** Number of DEGs in different pair-wise comparisons.

The DEGs between upper tepals and tepal bases at different developmental stages were identified and filtered with the R package DESeq (Anders and Huber, 2010) (Tables S4–S7). A total of 16,182 DEGs were detected in at least one pair-wise comparison (S2-D vs. S1-D, S2-D vs. S2-U, S3-D vs. S2-D, and S4-D vs. S3-D) (Figure 3A). Among the four comparisons, the largest number of DEGs was between the S2-D and S3-D libraries (9,958), with 4,775 down-regulated and 5,183 up-regulated unigenes (Figure 3B). The smallest number of DEGs was between the S2-D and S2-U libraries (2,036), with 671 down-regulated and 1,365 up-regulated unigenes (Figure 3B). Among the DEGs, 112 were significantly differentially expressed in all pair-wise comparisons (Figure 3A).

### Expression patterns of genes involved in anthocyanin biosynthesis

**Figure 4:**
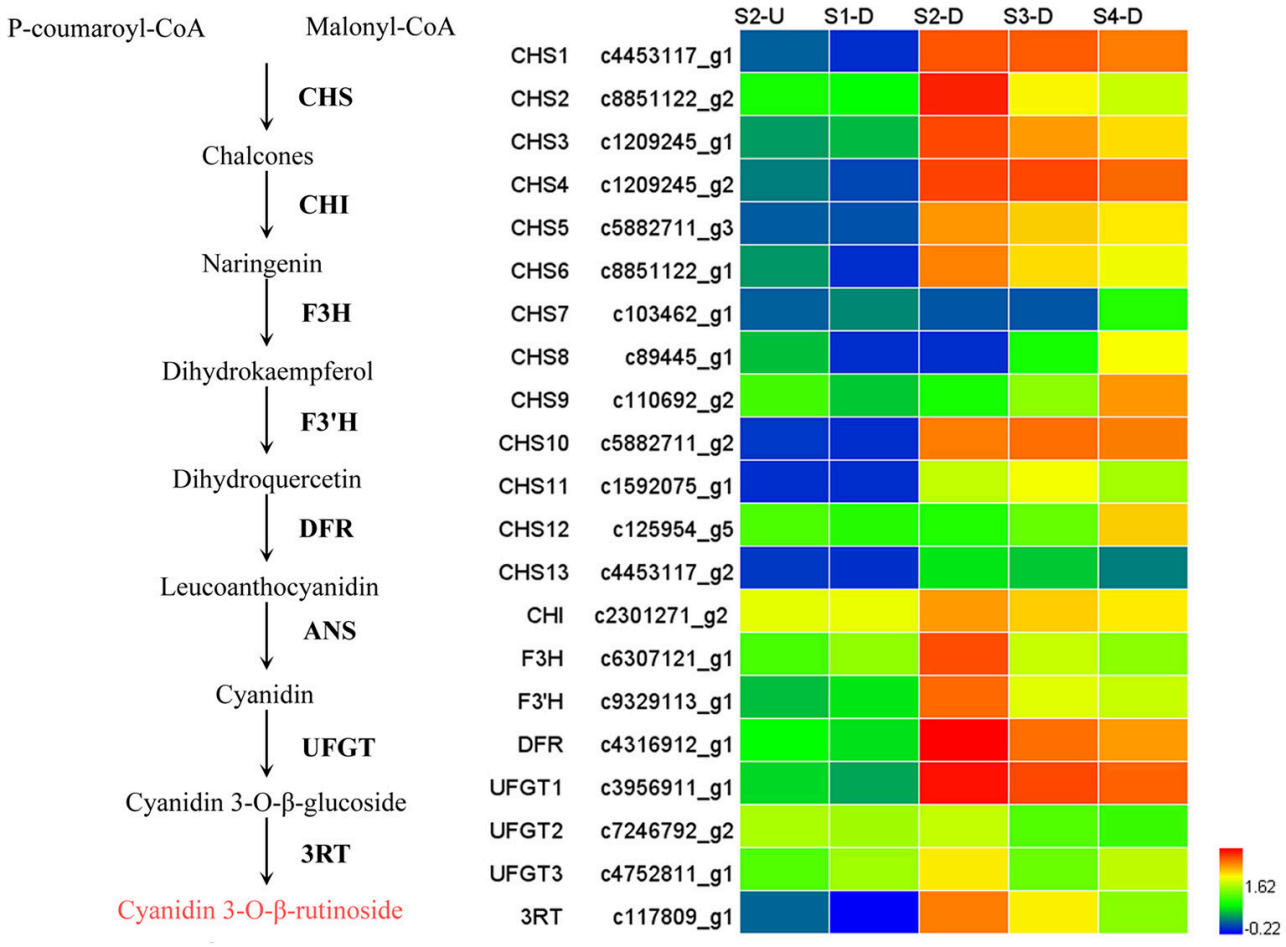
**Heatmap of differentially expressed genes (DEGs) related to anthocyanin biosynthesis**.

**Figure 5:**
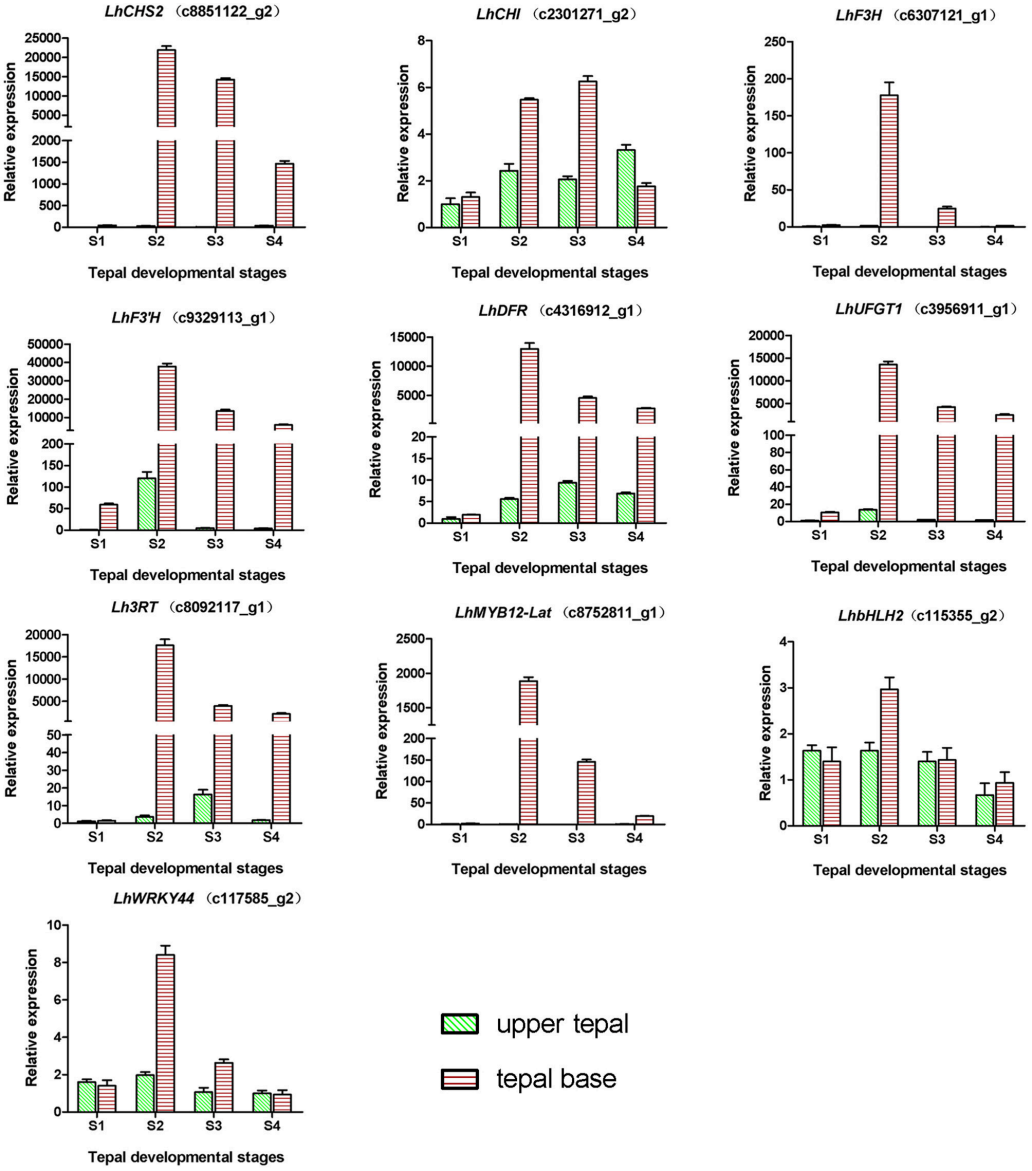
**Expression profiles of 10 unigenes related to anthocyanin biosynthesis in inner tepals of Asiatic “Tiny Padhye” during floral development**.

During bicolor tepal development in Asiatic “Tiny Padhye,” anthocyanins were heavily accumulated in the tepal bases but less so in the upper tepals. Therefore, we investigated genes encoding enzymes involved in anthocyanin biosynthesis. In this study, 61 unigenes encoding eight putative enzymes involved in anthocyanin biosynthesis were identified from the transcriptome (Table 2), and 21 of them were identified as DEGs (Table 2). As shown in Figure 4, most DEGs including *LhCHS1* (c4453117_g1), *LhCHS2* (c8851122_g2), *LhCHS3* (c1209245_g1), *LhCHS4* (c1209245_g2), *LhCHI* (c2301271_g2), *LhF3H* (c6307121_g1), *LhF3*′*H* (c9329113_g1), *LhDFR* (c4316912_g1), *LhUFGT1* (c3956911_g1), and *Lh3RT* (c8092117_g1) showed significantly higher expression in tepal bases at S2 and S3 than in the other samples, and extremely low expression in tepal bases at S1 and S4 and in upper tepals at S2. The results of qRT-PCR analyses not only confirmed these results, but also showed that anthocyanin structural genes had extremely low expression levels in upper tepals at all four stages (Figure 5).

### Expression patterns of genes involved in chlorophyll metabolism

**Figure 6:**
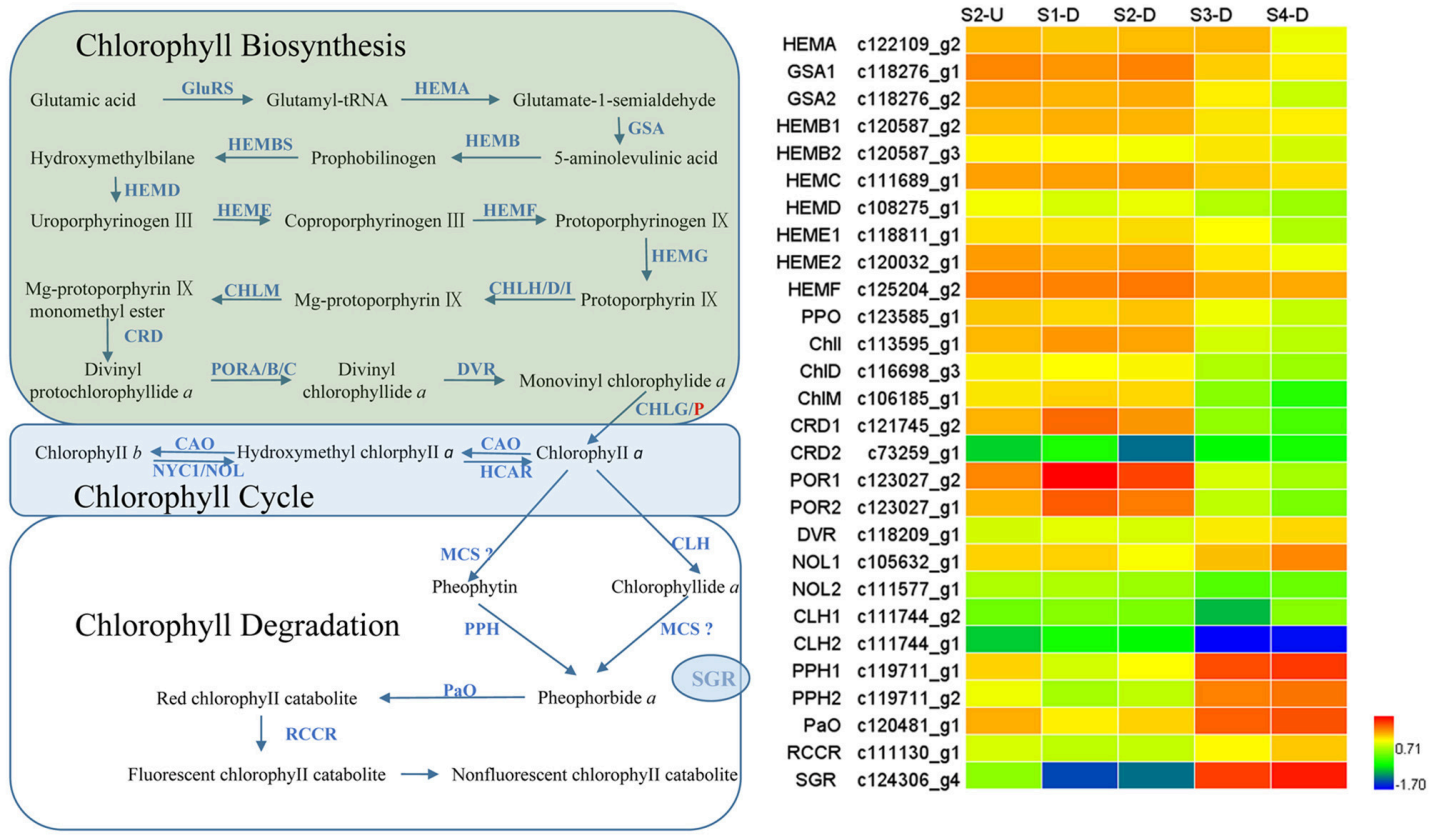
**Heatmap of differentially expressed genes (DEGs) related to chlorophyll metabolism**.

**Figure 7:**
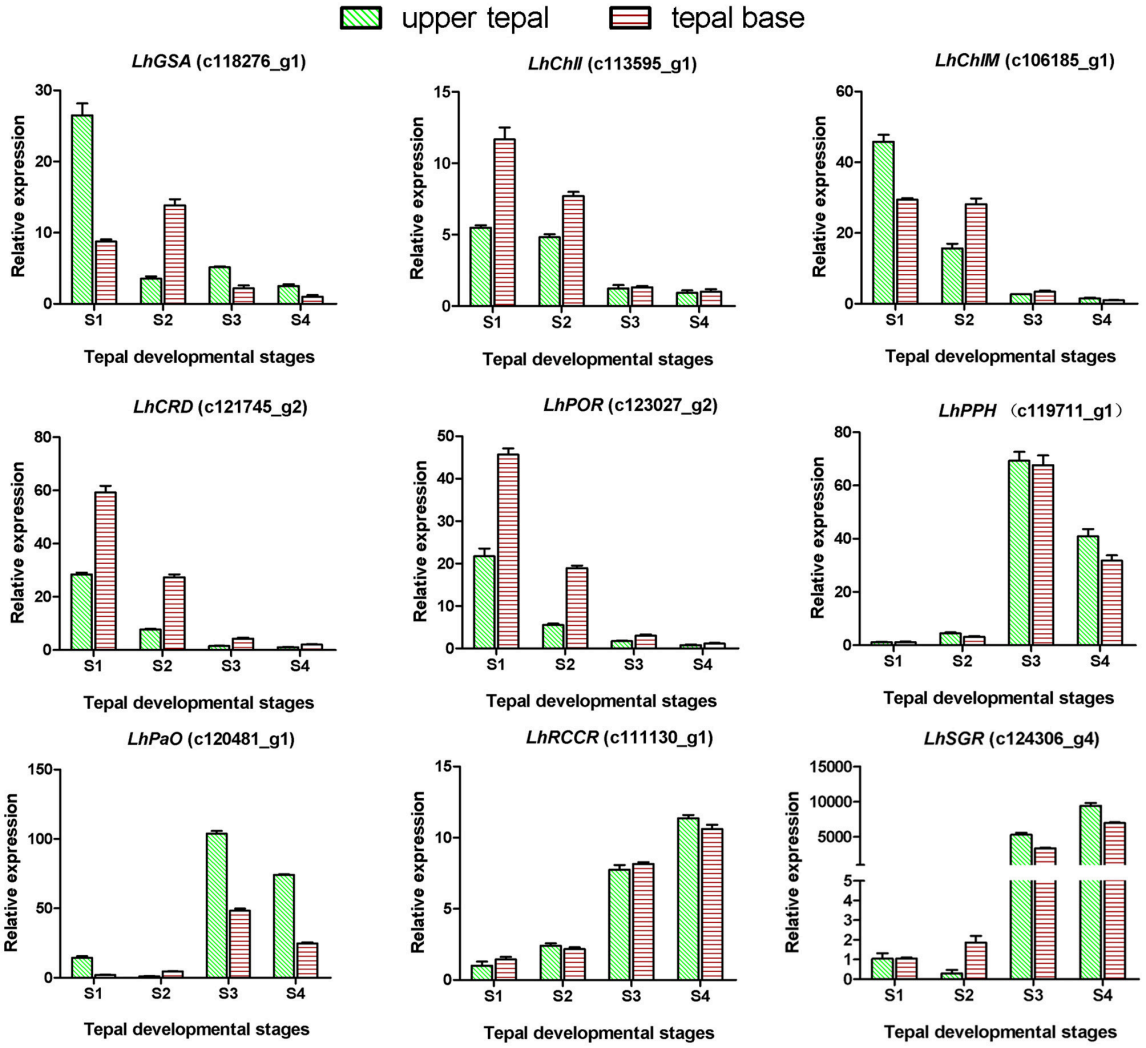
**Expression profiles of nine putative unigenes involved in chlorophyll metabolism in inner tepals of Asiatic “Tiny Padhye” during floral development**.

Degreening is a significant developmental change in the bicolor tepals of Asiatic “Tiny Padhye.” Therefore, we studied unigenes involved in Chl metabolism in detail. In this study, 106 candidate genes related to Chl metabolism were identified, and 28 of them were identified as DEGs (Table 2). These genes showed two distinct expression patterns (Figure 6). All of the DEGs related to Chl biosynthesis, except for *LhDVR* (c118209_g1), showed significantly higher expression in tepal bases at S1 and S2 and in the upper tepals at S2 than in the other stages. Their expression levels in tepal bases at S3 and S4 were extremely low (Figure 6). Conversely, most DEGs involved in Chl degradation showed the opposite pattern (Figure 6). The transcript levels of *LhPPH1* (c119711_g1), *LhPPH2* (c119711_g2), *LhPaO* (c120481_g1), *LhRCCR* (c111130_g1), and *LhSGR* (c124306_g4) were low at the early stages (S1 and S2) in the lower tepal parts, but high at the late stages (S3 and S4) (Figure 6). The results of qRT-PCR analyses not only confirmed these results but also showed that these DEGs shared similar expression patterns in upper parts during all four stages (Figure 7).

### Identification of WGCNA modules associated with anthocyanin biosynthesis and chlorophyll metabolism

**Figure 8:**
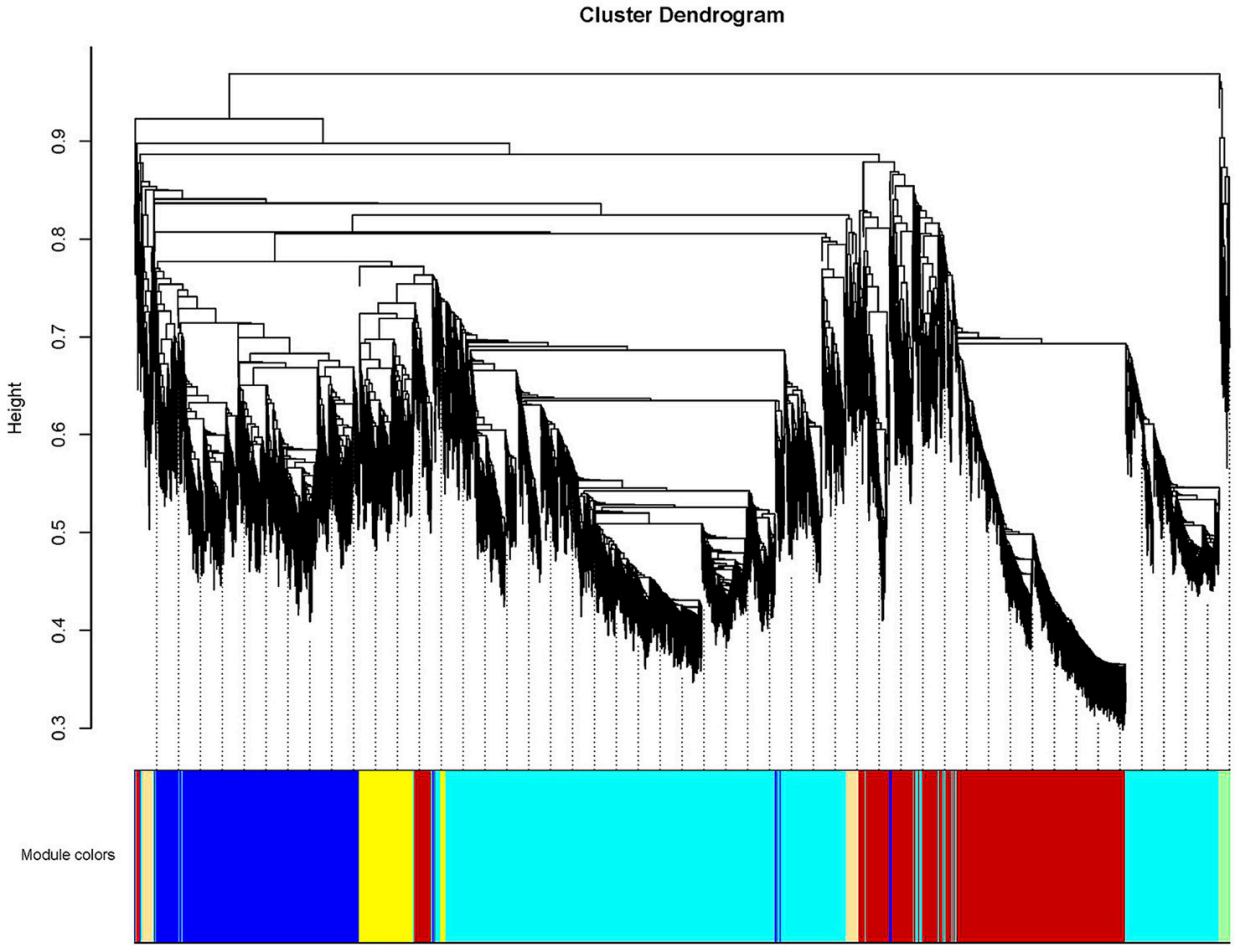
**Hierarchical cluster tree showing co-expression modules identified by weighted gene co-expression network analysis (WGCNA) of differentially expressed genes (DEGS)**. The dendrogram is produced by average linkage hierarchical clustering of 16,182 DEGS based on topological overlap of gene expression. The major tree branches constitute 26 distinct co-expression modules labeled by different colors underneath the dendrogram. Each of the 16,182 differentially expressed genes is represented by a leaf in the branch. Co-expression distance between two genes is shown as height on the y-axis.

To further investigate candidate genes related to anthocyanin biosynthesis and Chl metabolism during bicolor tepal development, co-expression gene network modules were constructed using WGCNA. The co-expression network constructed based on the 16,182 DEGs revealed 26 modules (Figure 8). In the dendrogram, each branch represents a module and each leaf constitutes one gene.

The Red module contained 12 out of 21 anthocyanin-related DEGs (Table 3), indicating that the 405 unigenes in the Red module play important roles in anthocyanin accumulation in lily bicolor tepals. The majority of the Chl biosynthesis-related DEGs were in the Yellow2 (9/19) module and more than half of the Chl degradation-related DEGs were in the Turquoise1 module (4/7) (Table 3). Therefore, the unigenes in the Yellow2 and the Turquoise1 modules were potentially involved in regulating Chl metabolism in lily tepals.

**Table 3:** **Distribution of differentially expressed genes (DEGs) and candidate structural genes related to anthocyanin biosynthesis and chlorophyll metabolism in 26 modules**.

**Module**	**DEGs (16,182)**	**DEGs related to anthocyanin biosynthesis (21)**	**DEGs related to chlorophyll biosynthesis (19)**	**DEGs related to chlorophyll degradation (7)**
	**No**.	**No**.	**No**.	**No**.
Red	405	12		
Turquoise2	2,254	4		1
Lightcyan1	41	2		
Brown1	1,343	1		
Cyan	174	1		
Midnightblue	159	1		
Blue1	1,827			1
Yellow2	1,723		9	
Turquoise1	2,494		4	4
Tan	205		3	
Brown2	336		1	
Pink	324		1	
Magenta	315		1	
Purple	236			1
Lightyellow	37			
Lightgreen	89			
Green	490			
Royalblue	35			
Gray	15			
Black	335			
Salmon	189			
Blue2	2,235			
Yellow1	519			
Gray60	110			
Lightcyan2	84			
Greenyellow	208			

### Construction of regulatory network

**Figure 9:**
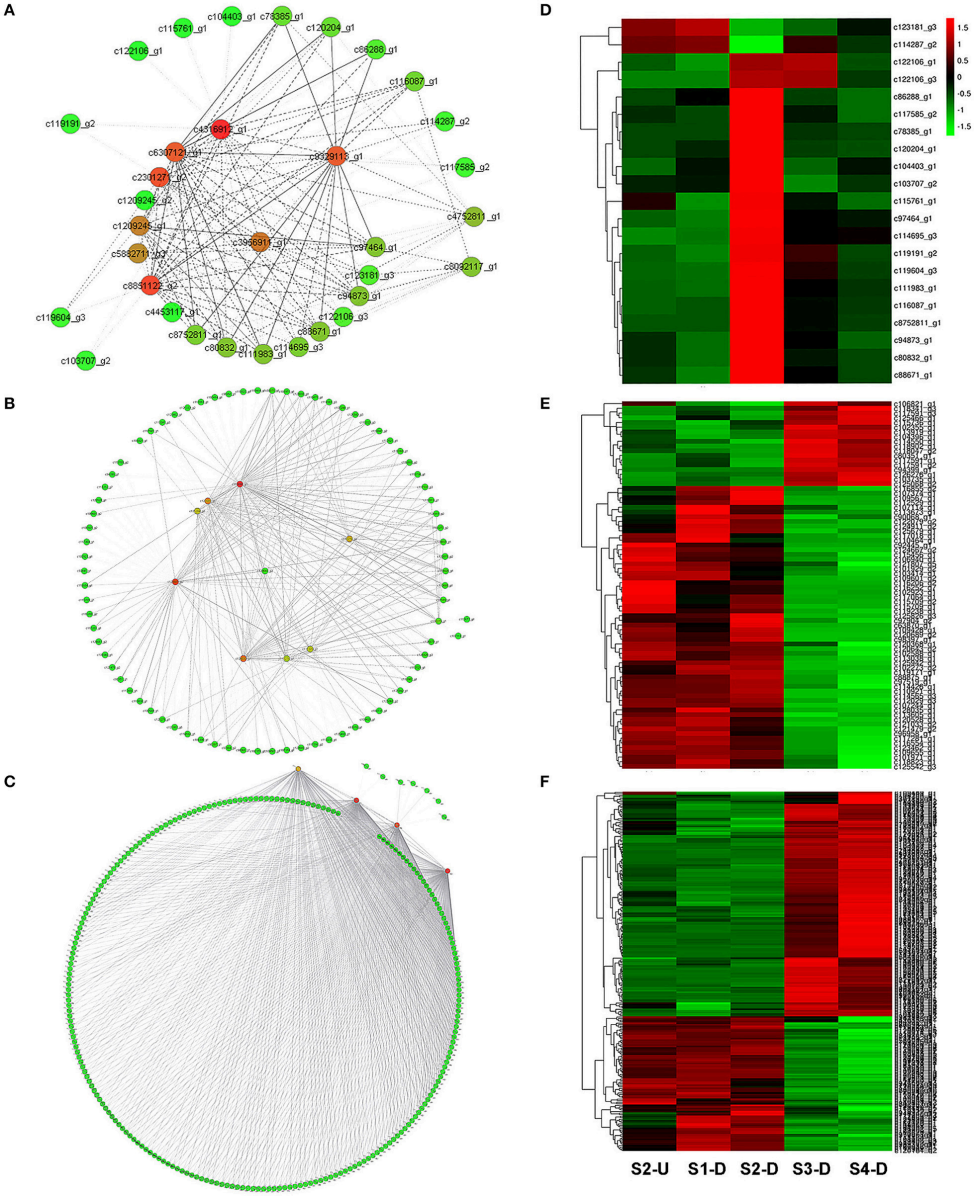
**Construction of regulatory networks of anthocyanin biosynthesis and chlorophyll metabolism and expression profiles of transcription factors (TFs). (A)** Subnetwork of putative TFs and structural genes related to anthocyanin biosynthesis from Red module. **(B)** Subnetwork of putative TFs and structural genes related to Chl biosynthesis from Yellow2 module. **(C)** Subnetwork of putative TFs and structural genes related to Chl degradation from Turquoise1 module. **(D)** Hierarchical clustering of expression profiles of 21 TFs related to anthocyanin biosynthesis. **(E)** Hierarchical clustering of expression profiles of 78 TFs related to Chl biosynthesis. **(F)** Hierarchical clustering of expression profiles of 232 TFs related to Chl degradation.

To provide a system view of the regulatory network responsible for controlling anthocyanin and Chl levels, networks were extracted from the Red, Yellow2, and Turquoise1 modules. To search for regulatory genes potentially involved in anthocyanin biosynthesis in lily tepals, we constructed a subnetwork from the Red module using 12 anthocyanin biosynthesis-related genes as the seed nodes. As a result, 12 anthocyanin genes and 21 TFs belonged to this subnetwork (Figure 9A and Table 4). These TFs were classified into 10 putative TF families, with the four largest TF families being the MYB (three unigenes), WRKY (three unigenes), Dof (three unigenes), and ERF (three unigenes) families (Table 4 and Table S8). The expression profiles of these TFs potentially related to anthocyanin biosynthesis were hierarchically clustered and plotted in a heatmap (Figure 9D). The expression patterns of all 21 TFs, except for c123181_g3 and c114287_g2, were positively correlated with those of anthocyanin biosynthetic genes.

**Table 4:** **Categorization of putative transcription factors (TFs) in Red, Yellow2, and Turquoise1 modules**.

**TF family**	**Red module**	**Yellow2 module**	**Turquoise1 module**	**TF family**	**Red module**	**Yellow2 module**	**Turquoise1 module**
AP2	0	2	1	HB-other	0	1	1
ARF	0	1	5	HD-ZIP	1	2	2
B3	0	1	3	HSF	0	0	3
bHLH	2	21	16	LBD	1	0	3
BBR-BPC	0	0	1	LSD	0	0	1
bZIP	0	3	8	MIKC	0	2	0
C2H2	0	5	14	MYB	3	6	2
C3H	0	5	13	NAC	0	4	29
CAMTA	0	0	2	NF-X1	0	1	1
CO-like	2	2	5	NF-YA	0	0	1
DBB	1	0	0	NF-YB	0	1	3
Dof	3	0	1	MYB related	2	2	5
ERF	3	3	11	SBP	0	1	2
FAR1	0	0	3	TALE	0	0	3
G2-like	0	0	3	TCP	0	4	1
GATA	0	2	2	Trihelix	0	3	9
GeBP	0	1	2	Whirly	0	1	0
GRAS	0	1	6	WRKY	3	3	65
GRF	0	0	1	ZF-HD	0	0	4

Using these Chl biosynthesis-related genes as bait, a huge subnetwork was extracted from the Yellow2 module. This subnetwork contained nine Chl biosynthesis-related genes and 78 TFs belonging to 25 TF families (Figure 9B and Table 4). Among the TF families, bHLH (21 genes) was the largest group, followed by MYB (six genes), C2H2 (five genes), and C3H (five genes) (Table 4 and Table S9). Among the TFs, 60 had expression patterns that were positively correlated with Chl content and the remainder had expression patterns that were negatively correlated with Chl content (Figure 9E).

In the Turquoise1 module, four Chl degradation-related unigenes were highly interconnected and 232 TFs, belonging to 35 different families, had interactions with these Chl degradation-related unigenes (Figure 9C and Table 4). The main TF family was the WRKY family (65 unigenes), followed by the NAC TF family (29 unigenes), and the bHLH family (16 unigenes) (Table 4 and Table S10). Notably, these TFs also showed two distinct expression patterns (Figure 9F).

## Discussion

### Time-series transcriptome and co-expression network analysis of lily bicolor tepal development

To date, two previous studies have identified anthocyanin biosynthetic and regulatory genes in lily flowers based on transcriptome analysis by RNA-seq (Zhang et al., 2015; Suzuki et al., 2016). However, those studies were limited by the small number of samples used and the small scale of transcriptome data obtained. For example, to investigate the molecular mechanisms responsible for bicolor tepal development in lilies, Suzuki et al. (2016) analyzed the global transcription of pigmented and non-pigmented tepal parts from “Lollypop” at stage 3 with two replicates. This resulted in ~50 million raw reads and a total of 39,426 unigenes through *de novo* assembly. However, as that study did not provide a global view of transcriptome dynamics over the key tepal developmental stages of anthocyanin biosynthesis, it was hard to perform a co-expression network analysis to identify new candidate target genes. Our transcriptome profiling and network analysis differ from these prior studies in several ways. First, we not only analyzed the global transcription of pigmented and non-pigmented tepal parts from “Tiny Padhye” at stage 2 but also constructed a high-resolution transcriptome atlas of lily inner tepal development using a time series of tepal base samples taken from S1 to S4. Thus, ~772.62 million raw reads were generated and 295,787 unigenes were assembled. This large-scale transcriptome analysis serves as a valuable resource for analyzing gene function on a global scale, and for elucidating the developmental processes of anthocyanin biosynthesis and Chl metabolism. Second, all the genes involved in Chl metabolism were identified in *Lilium* spp. using the KEGG analysis, and their expression profiles during tepal development were analyzed to identify DEGs. Additionally, using WGCNA, a number of candidate TFs involved in anthocyanin biosynthesis and Chl metabolism were identified in lilies. Overall, with multiple time-points and a co-expression analysis, we can say that the present work is the first dynamic transcriptomic study of lily flower color development.

### Regulatory network of anthocyanin biosynthesis in lily bicolor tepals

In this study, we identified 61 unigenes encoding eight candidate enzymes in the anthocyanin biosynthetic pathway from the lily transcriptome. Among these unigenes, 21 were DEGs and most of them were expressed at high levels coordinately in tepal bases, while showing extremely low expression levels in the upper tepals at each stage. Similar expression patterns have been reported for “Lollypop” tepals (Suzuki et al., 2016). These results indicate that the bicolor trait of “Tiny Padhye” is caused by the transcriptional regulation of anthocyanin biosynthesis genes rather than the PTGS of *CHS* genes.

Many studies have demonstrated the crucial role of TFs in regulating anthocyanin biosynthesis in plants. In this study, we identified 10 TF families comprising 21 TF unigenes that represented potentially important regulators of anthocyanin biosynthesis in *Lilium* spp. The R2R3-MYB family plays a key role in regulating the spatiotemporal expression of anthocyanin biosynthetic genes in plants (Gonzalez et al., 2008; Zhao and Tao, 2015). This family is further classified into several subgroups, with the members of subgroup 6 positively regulating anthocyanin biosynthesis (Dubos et al., 2010). In this study, the sequence of c8752811_g1, designated as a subgroup 6 R2R3-MYB, was found to be the same as that of *LhMYB12-Lat*, which activates anthocyanin accumulation in lily tepals (Yamagishi et al., 2014). This result shows that the WGCNA is a powerful method of identifying TFs relevant to a specific pathway. Several anthocyanin repressors have been characterized, including small R3-MYBs, AtMYBL2, and subgroup 4 R2R3-MYBs. Some R3-MYBs [CAPRICE (CPC) from *A. thaliana*, and MYBx from petunia (*P. hybrida*)] contain a single MYB DNA-binding domain (DBD) and a conserved amino acid motif ([DE]Lx2[RK]x3Lx6Lx3R) required to bind bHLH partners, and are found to regulate anthocyanin biosynthesis by competing with R2R3-MYB activators for interaction with bHLH partners (Zhu et al., 2009; Albert et al., 2014). AtMYBL2 from *Arabidopsis* lacks an intact R2-MYB repeat, and contains a repression motif (TLLLFR) to actively repress transcription (Dubos et al., 2008; Matsui et al., 2008). Subgroup 4 R2R3-MYBs [Fa/Fc-MYB1 from strawberry (*Fragaria* spp.) and Ph MYB27 from petunia (*P. hybrida*)] contain an ERF-associated amphiphilic repression (EAR) motif that is essential for the repressive function (Aharoni et al., 2001; Salvatierra et al., 2013; Albert et al., 2014). The unigene c120204_g1(*LhMYB3*) was designated as a subgroup 4 R2R3-MYB, suggesting that this MYB suppressor might be involved in bicolor development. We also identified one unigene (c115761_g1), *LhMYBP*, as a homolog of *ZmP*. In maize, ZmP activates the promoters of *CHS, CHI, F3H*, and *FLS*, but it does not control anthocyanin accumulation (Grotewold et al., 1994; Mehrtens et al., 2005). Thus, the function of *LhMYBP* needs to be further studied. In our study, three putative WRKY TFs were identified including the unigene (c117585_g2) *LhWRKY44* that strongly attracted our attention. *AtTTG2* (*WRKY44*) and its homologs have different functions in different species. In *Arabidopsis, AtTTG2* is involved in trichome initiation, proanthocyanidin accumulation in the seed coat, and seed development (Johnson, 2002; Ishida et al., 2007; Dilkes et al., 2008). However, in *Brassica napus, Bna.TTG2* genes, homologs of *AtTTG2* (*WRKY44*), are involved in the salt stress response (Li et al., 2015). In *P. hybrida, PH3*, which is homologous to *AtTTG2* (*WRKY44*), regulates color patterns by acidifying the central vacuole where anthocyanins are stored in epidermal petal cells (Verweij et al., 2016). In the present study, the expression pattern of *LhWRKY44* was parallel to those of anthocyanin biosynthetic genes, but its function is still unknown in *Lilium* spp. and requires further research.

### Regulatory network of chlorophyll metabolism in lily bicolor tepals

**Figure 10:**
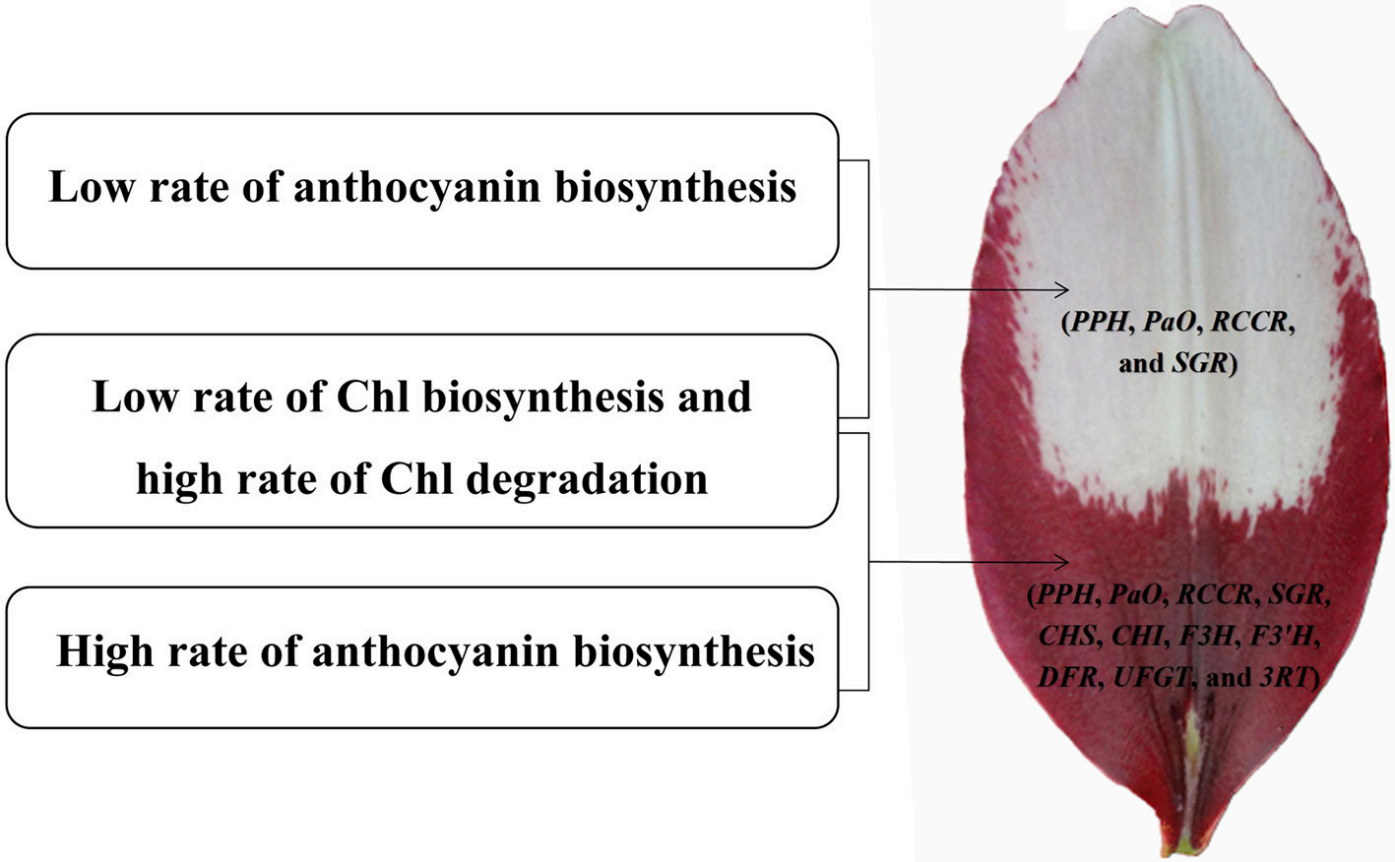
**Model of molecular mechanisms of (white and purple) bicolor lily tepal development**. Four genes involved in Chl degradation pathway (*PPH, PaO, RCCR*, and *SGR*) are highly expressed in the white upper tepals of Asiatic “Tiny Padhye”. Four genes involved in Chl degradation pathway (*PPH, PaO, RCCR*, and *SGR*) and seven genes involved in anthocyanin biosynthetic pathway (*CHS, CHI, F3H, F3*′*H, DFR, UFGT*, and *3RT*) are highly expressed in the purple basal tepals.

We identified 106 candidate structural genes involved in Chl metabolism in the transcriptome dataset, and 28 of these unigenes were identified as DEGs. The expression of most DEGs in Chl biosynthesis declined at S3 and S4, correlating strongly with Chl content. The expression pattern of most DEGs involved in Chl degradation was opposite to that of DEGs involved in Chl biosynthesis. Overall, based on the gene expression and Chl content analysis, our results indicate that high-level expression of Chl degradation genes and low-level expression of Chl biosynthetic genes lead to the absence of Chl from “Tiny Padhye” tepals after flowering (Figure 10).

TFs are crucial regulatory proteins that mediate transcriptional regulation. Many previous studies have identified the TFs responsible for controlling Chl metabolism in leaves (Lim, 2003; Lin et al., 2015; Wen et al., 2015). Three TFs, ANAC046, ETHYLENE INSENSITIVE3, and ORE1, positively regulate Chl degradation by binding to the promoter regions of *NYC, SGR1*, and *PaO* (Qiu et al., 2015; Oda-Yamamizo et al., 2016). FLU, a negative regulator of Chl biosynthesis in *A. thaliana*, may mediate its regulatory effect through interaction with enzymes (GSA and CHlH) involved in Chl biosynthesis (Meskauskiene et al., 2001). However, the molecular mechanisms regulating Chl metabolism in lily petals are still unknown. In this study, we identified 78 TFs in the Chl biosynthesis co-expression network. These TFs were categorized into 25 putative TF families (Table S9). The main TF family was the bHLH family (21 unigenes), followed by the MYB family (six unigenes), the C2H2 family (five unigenes), and the C3H family (five unigenes). These results indicate that there is a complicated regulatory network controlling Chl biosynthesis in lily tepals.

Several TF families, especially the NAC and WRKY families, have members that control Chl degradation in leaves (Lim, 2003; Wen et al., 2015). Here, we found 94 TFs belonging to these families in the Chl degradation co-expression network (Table 4; Table S10). This result indicated that, in both leaves and tepals, some members of the NAC and WRKY families have highly conserved functions in regulating Chl degradation. An additional 26 TF families (bHLH, C2H2, C3H, and others) were also detected in this study.

We further analyzed the expression patterns of these TFs, and observed two distinct expression patterns for TFs involved in both Chl biosynthesis and Chl degradation. The expression profiles of some TFs were positively correlated with Chl content, while those of other TFs were negatively correlated with Chl content, suggesting that both transcriptional activation and repression are involved in regulating Chl biosynthesis and Chl degradation in lily tepals.

In this study, we presented a dynamic transcriptome landscape of lily bicolor tepal development using RNA-seq technology. Based on *de novo* transcriptome analysis and functional annotation of the transcriptome dataset, we identified transcripts encoding most of the known enzymes involved in the anthocyanin biosynthetic and Chl metabolic pathways. Our results indicate that the white upper tepals are caused by a low rate of Chl biosynthesis, a high rate of Chl degradation, and a low rate of anthocyanin biosynthesis. The purple tepal bases are due to a high rate of anthocyanin biosynthesis, a low rate of Chl biosynthesis, and a high rate of Chl degradation (Figure 10). We identified regulatory genes that are likely to be key regulators of anthocyanin biosynthesis and Chl metabolism using WGNCA. Taken together, these results pave the way for the greater understanding of the molecular basis of lily bicolor tepal development and will allow for the identification of candidate genes associated with anthocyanin biosynthesis and Chl metabolism in *Lilium* spp.

## Author contributions

JM designed the research. LX, PY, SY, YF, HX, YT, YC, and XL conducted the experiments. LX analyzed the data and wrote the manuscript.

### Conflict of interest statement

The authors declare that the research was conducted in the absence of any commercial or financial relationships that could be construed as a potential conflict of interest.
