# Supplementary material for: Spatiotemporal Transcriptome Analysis Provides Insights into Bicolor Tepal Development in Lilium “Tiny Padhye”
Source: Front Plant Sci. 2017 Mar 24;8:398. doi: 10.3389/fpls.2017.00398 (PMC5364178; doi:10.3389/fpls.2017.00398)
Supplement: Supplementary file 11 [file Presentation1.PDF]

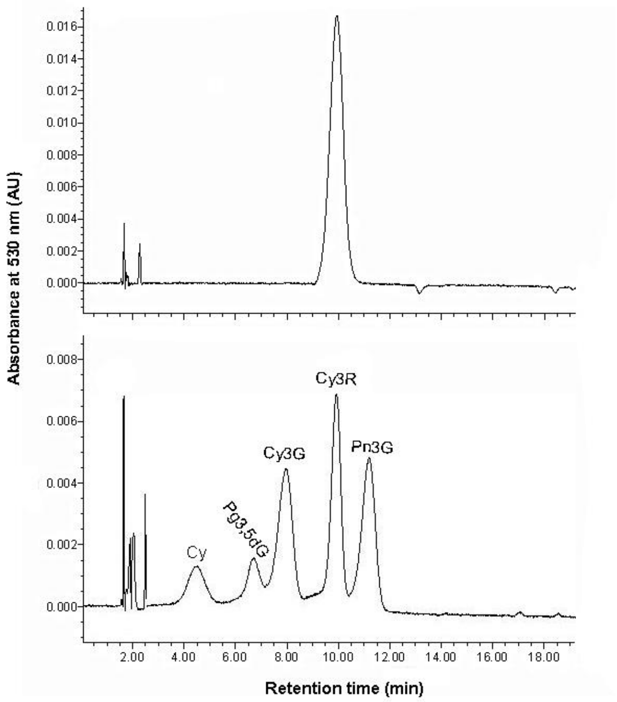

Figure S1. HPLC chromatograms of products from tepal bases of Asiatic 'Tiny Padhye' at stage 3 (S3). **(A)** HPLC chromatograms of anthocyanins extracted from tepal bases of Asiatic 'Tiny Padhye' at S3. **(B)** HPLC chromatograms of a mixture of authentic cyanidin (Cy), pelargonidin 3,5-diglucoside (Pg3,5dG), cyanidin 3-O-β-glucoside (Cy3G), cyanidin 3-O-β-rutinoside (Cy3R), and peonidin 3-O-β-glucoside (Pn3G).
